# The Effect of Elevated Alanine Transaminase on Non-invasive Prenatal Screening Failures

**DOI:** 10.3389/fmed.2022.875588

**Published:** 2022-06-15

**Authors:** Ping Chen, Longwei Qiao, Sheng Zhang, Jieyu Jin, Jun Cao, Yuqiong Zhang, Haoyu Tang, Zheng Yu, Jingye Shi, JingPing Yin, Yuting Liang, Xiao Wu

**Affiliations:** ^1^Department of Obstetrics, School of Gusu, The Affiliated Suzhou Hospital of Nanjing Medical University, Suzhou Municipal Hospital, Nanjing Medical University, Suzhou, China; ^2^Center for Reproduction and Genetics, School of Gusu, The Affiliated Suzhou Hospital of Nanjing Medical University, Suzhou Municipal Hospital, Nanjing Medical University, Suzhou, China; ^3^Center for Clinical Laboratory, The First Affiliated Hospital of Soochow University, Suzhou, China; ^4^Department of Medical Laboratory, The Affiliated Suzhou Hospital of Nanjing Medical University, Suzhou Municipal Hospital, Nanjing Medical University, Suzhou, China

**Keywords:** non-invasive prenatal screening, fetal fraction, cell-free DNA, ALT, multivariable linear regression models

## Abstract

**Objective:**

To determine the effects of alanine transaminase (ALT) levels on the screening failure rates or “no calls” due to low fetal fraction (FF) to obtain a result in non-invasive prenatal screening (NIPS).

**Methods:**

NIPS by sequencing and liver enzyme measurements were performed in 7,910 pregnancies at 12–26 weeks of gestation. Univariate and multivariable regression models were used to evaluate the significant predictors of screening failure rates among maternal characteristics and relevant laboratory parameters.

**Results:**

Of the 7,910 pregnancies that met the inclusion criteria, 134 (1.69%) had “no calls.” Multiple logistic regression analysis demonstrated that increased body mass index, ALT, prealbumin, albumin levels, and *in vitro* fertilization (IVF) conception rates were independently associated with screening failures. The test failure rate was higher (4.34 vs. 1.41%; *P* < 0.001) in IVF pregnancies relative to those with spontaneous conceptions. Meanwhile, the screening failure rates increased with increasing ALT levels from 1.05% at ≤10 U/L to 3.73% at >40 U/L. In particular, IVF pregnancies with an ALT level of >40 U/L had a higher test failure rate (9.52%). Compared with that for an ALT level of ≤10 U/L, the adjusted odds ratio of “no calls” for ALT levels of 10–20, 21–40, and >40 U/L was 1.204 [95% confidence interval (CI), 0.709–2.045], 1.529 (95% CI, 0.865–2.702), and 2.764 (95% CI, 1.500–5.093) (*P*_*trend*_ < 0.001), respectively.

**Conclusions:**

Increased ALT and IVF conceptions were associated with a higher screening failure rates in NIPS. Therefore, a feasible strategy to adjust these factors to reduce the probability of “no calls” due to low FF would be of great clinical significance.

## Introduction

Sequencing of cell-free DNA (cfDNA) in maternal plasma for the detection of fetal trisomies 21, 18, and 13, with unprecedented sensitivity and specificity (1, 2), is an attractive method for non-invasive prenatal screening (NIPS); however, the low fetal fraction (FF) (<4%) of cfDNA usually results in screening failures or “no calls” (3, 4). The reasons for NIPS test failure are low FF, insufficient plasma, hemolysis, quality control failure, excessive cfDNA concentration, and Z value in the gray area. The low FF may be responsible for up to 50% of all the failures (5, 6). Therefore, numerous researchers have studied the effects of maternal-fetal characteristics and experimental factors on the FF, with the aim of adjusting these factors to obtain adequate fetal-derived cfDNA to reduce the probability of screening failures. Multiple factors, including maternal body mass index (BMI), *in vitro* fertilization (IVF)-conceived pregnancies, gestational age (GA), anticoagulation therapy, medication intake, blood collection and sample transportation to the laboratory, triglyceride (TG) levels, and fetal aneuploidy, have been demonstrated to be associated with low FF and test failure rate (7–12). Identifying which candidate factors are associated with test failure during cfDNA testing due to insufficient FF remains a challenge.

The cfDNA fragments in a pregnant woman's plasma are derived from three tissues of origin: the placenta, maternal-derived DNA, and fetus (or fetuses) (13). Maternal-derived DNA is a major source of cfDNA in maternal plasma; it is primarily derived from white blood cells that release cfDNA during cell turnover. However, a large variety of solid organs, such as the liver, adipose tissue, and stromal vascular tissues, also contribute to the circulating plasma pool (14, 15). Indeed, some clinical data have confirmed that certain maternal medical conditions, including intrahepatic cholestasis of pregnancy (16), vitamin B12 deficiency (17), and autoimmune diseases (18–22), can lead to NIPS testing failures. However, liver-derived cfDNA is the main source of circulating DNA pool, and studies examining whether changes in liver function affect NIPS test failures remain scarce.

To the best of our knowledge, a similar evaluation of the parameters affecting NIPS testing failures has not been published to date. This study assessed 7,910 pregnancies (at 12–26 weeks of gestation) wherein NIPS by sequencing, pregnancy patterns (IVF or spontaneous conceptions), and liver enzymes measurements were performed. We aimed: (1) to investigate the potential role of maternal-fetal characteristics and liver biochemical parameters in predicting NIPS testing failures and (2) to evaluate if adjusting these factors could be useful for the clinical management of these pregnancies in cases with low FF.

## Materials and Methods

### Study Population

This was a single-center, retrospective cohort study. Data for a total of 7,910 pregnancies were collected at Suzhou Municipal Hospital between January 2016 and December 2020 after approval by the Reproductive Medicine Ethics Committee of Suzhou Municipal Hospital (ID: K-2021-032-H01). This ethics approval is to study the effect of maternal characteristics on NIPT test failures, as described in our previous study (12). The data for this study were derived from pregnant women who underwent NIPS for trisomies 21, 18, 13, and in whom serum liver biochemical parameters were analyzed at 12–26 weeks of gestation. All the pregnant women had their Hepatitis B surface antigen (HBsAg) test records. The exclusion criteria included minors (<18 years of age), vanishing twins, preference for immediate invasive testing, and poor blood collection.

### Procedure and Data Collection

Serum samples of all the pregnant women were collected after an overnight fasting. Biochemical parameters, including total bile acid (TBA), albumin/globulin ratio (A/G), albumin, globulin, total bilirubin (TBIL), indirect bilirubin (IBIL), direct bilirubin (DBIL), prealbumin (PAB), total protein (TP), enzyme aspartate aminotransferase (AST), alanine aminotransferase (ALT), alkaline phosphatase (ALP), γ-glutamyl transpeptidase (γ-GT), lactate dehydrogenase (LDH), cholinesterase (chE), and fasting blood glucose (FBG), were determined using Beckman Coulter AU5800 (Beckman Coulter, Brea, CA, USA) according to the manufacturer's instructions.

All the patients received pretest counseling, and informed consent was obtained by genetic counselors before NIPS testing. Genetic counseling mainly included older pregnant women, previous pregnancy affected by fetal aneuploidy, and ultrasonic detection of aneuploidy or fetal abnormalities. Women using NIPS underwent ultrasonography before 14 weeks of gestation to determine the number of fetuses, chorionicity, GA, maternal and fetal characteristics, and medical history, including maternal age, weight, BMI, singleton or twin pregnancies, and method of conception.

Blood samples (10 mL) were collected on-site from consenting participants following pretest counseling provided by the genetic counselors. Maternal plasma cfDNA was isolated using the QIAamp DSP DNA Blood Mini Kit (Qiagen) according to the manufacturer's instructions. The cfDNA library was constructed using polymerase chain reaction (PCR). Sequencing libraries were sequenced using the Ion Proton system or the BGISEQ-500 (MGI, China) system. The FF for the pregnancies was assessed by calculating the proportion of chromosome Y reads for male fetuses, and by using FF- QuantSC, which uses an artificial neural network model (FF-QuantSC: accurate quantification of fetal fraction by a neural network model), for female fetuses. A low FF, usually <4%, can result in a “no call.” All the samples had a fetal karyotype (NIPS-positive pregnancy) or clinical follow-up results.

### Statistical Analyses

The main outcomes were testing failures of NIPS and liver biochemical parameters. Secondary outcomes were the FF in different ALT groups, IVF conceptions, and spontaneous conceptions.

Descriptive data are expressed as medians and interquartile ranges (IQR) for non-normally distributed continuous variables and as absolute values and percentages for categorical variables. Univariate analysis and multivariable logistic regression models were used to examine the associations of the rate of NIPS testing failures (FF <4%) with liver biochemical parameters and adjustment for confounders (i.e., BMI). We selected the confounders based on their associations with the outcomes of interest or a 10% change in the effect estimate.

ALT levels were categorized as ≤10, 10–20, 21–40, and >40 U/L according to the optimal scaling. We used three different models to analyze the impact of each ALT category on the test failure. Model 1 was a univariate logistic regression analysis of the relationship between the ALT level and test failure rate. Model 2 was adjusted for BMI, PAB, and albumin levels. Model 3 was adjusted for IVF conception rates plus the variables included in Model 2. The sample size for regression analysis was estimated by the events per variable (EPV) method (23). Data analysis was performed using SPSS version 26.0 (IBM Corp, Armonk, NY, USA). All *P-*values were two-sided, and statistical significance was set at *P* < 0.05.

## Results

### Sample Characteristics

Based on the selection criteria described above, 7,910 pregnancies with NIPS results and liver biochemical parameters were included in the analysis. Of these, 7,149 (90.4%) pregnancies were conceived spontaneously and 761 (9.6%) were achieved by IVF.

The sample characteristics and relevant laboratory parameters of the study population are listed in Table 1. The median GA was 17 weeks (16–18 weeks), and the median maternal BMI was 22.30 kg/m^2^. Of these samples, the median FF was 10.22%, and 1.7% of the samples had an FF of <4%. Moreover, the median ALT level was 15 (11–23) U/L, and 8.5% of the samples had an ALT level of >40 U/L.

**Table 1:** Sample characteristics of the study population (*n* = 7,910).

**Characteristic**	**Value (median and interquartile range)**
Gestational age (week)	17 (16–18)
FF (%)	10.22 (8.02–12.89)
BMI (kg/m^2^)	22.30 (20.55–24.38)
TBA(μmol/L)	1.40 (0.90–2.30)
A/G	1.50 (1.40–1.70)
TBIL(μmol/L)	8.30 (6.90–10.30)
DBIL (μmol/L)	1.90 (1.50–2.40)
IBIL (μmol/L)	6.40 (5.30–8.00)
PAB (mg/L)	231.50 (212.00–252.30)
TP (g/L)	72.50 (69.90–75.30)
Albumin (g/L)	44.00 (42.10–46.10)
Globulin (g/L)	28.50 (26.20–30.70)
AST (U/L)	18 (15–22)
ALT (U/L)	15 (11–23)
ALP (U/L)	53 (46–61)
γ-GT (U/L)	14 (11–19)
LDH (U/L)	165 (148–182)
ChE (U/L)	291 (258–328)
FBG (mmol/L)	4.59 (4.34–4.87)

*FF, fetal fraction; TBA, total bile acid; A/G, albumin /globulin ration; TBIL, total bilirubin; IBIL, indirect bilirubin; DBIL, direct bilirubin; PAB, prealbumin; TP, total protein; AST, enzyme aspartate aminotransferase; ALT, alanine aminotransferase; ALP, alkaline phosphatase; γ-GT, γ-glutamy transpeptidase; LDH, lactate dehydrogenase; chE, cholinesterase; FBG, fasting blood glucose*.

### Multiple Factors Affecting CfDNA Test Failures

**Figure 1:**
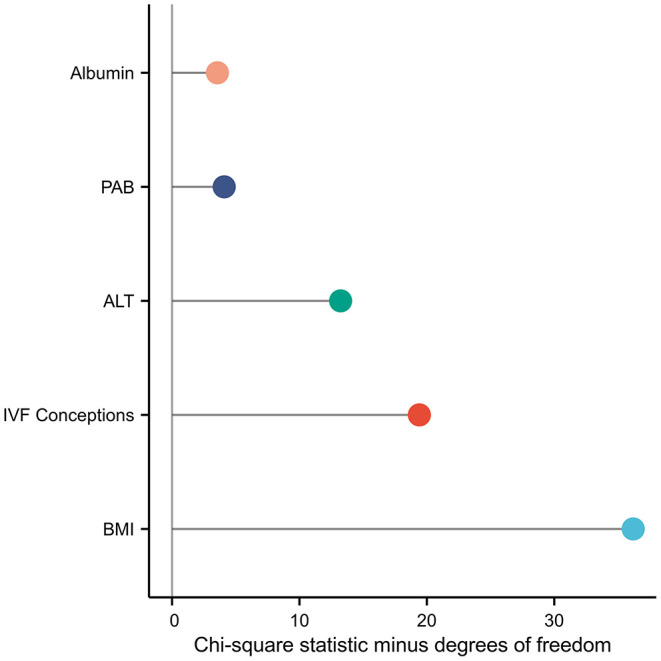
Each factor's contribution in the multivariable logistic regression analysis was measured as the chi-square statistic minus the degree of freedom of the predictor.

Among the 7,910 pregnancies that met the inclusion criteria, 134 (1.69%) had “no calls.” Univariate regression analysis demonstrated that the test failure rate was positively correlated with the IVF conception [odds ratio (OR), 3.163; 95% confidence interval (CI), 2.120–4.721; *P* < 0.001]; BMI (OR, 1.163; 95% CI, 1.113–1.215; *P* < 0.001); and TBA (OR, 1.061; 95% CI, 1.004–1.122; *P* = 0.035), PAB (OR, 1.010; 95% CI, 1.005–1.015; *P* < 0.001), AST (OR, 1.011; 95% CI, 1.005–1.017; *P* < 0.001), ALT (OR, 1.007; 95% CI, 1.004–1.010; *P* < 0.001), γ-GT (OR, 1.019; 95% CI, 1.010–1.028; *P* < 0.001), LDH (OR, 1.004; 95% CI, 1.000–1.008; *P* = 0.0425), chE (OR, 1.003; 95% CI, 1.000–1.006; *P* = 0.029), and FBG levels (OR, 1.750; 95% CI, 1.334–2.297; *P* < 0.001) (Table 2). Moreover, the multivariable logistic regression analysis demonstrated that the risk of test failure increased with increasing BMI (OR, 1.151; 95% CI, 1.100–1.204; *P* < 0.001), ALT (OR, 1.007; 95% CI, 1.004–1.011; *P* < 0.001), PAB (OR, 1.006; 95% CI, 1.001–1.011; *P* = 0.024), albumin (OR, 1.067; 95% CI, 1.005–1.133; *P* = 0.033) levels, and IVF conception (OR, 2.604; 95% CI, 1.719–3.944; *P* < 0.001) (Table 2). Each factor's contribution in the multivariable logistic regression analysis was measured as the chi-square statistic minus degrees of freedom of the predictor. Results indicate that the IVF conception rate and ALT level are important factors affecting test failure, in addition to BMI (Figure 1).

**Table 2:** Regression analysis of factors from maternal characteristics biochemical parameters for predicting FF in 7,910 pregnancies.

	**Univariate analysis**		**Multivariable analysis**	
**Independent variable**	**Odds ratio (95% CI)**	***P*-value**	**Odds ratio (95% CI)**	***P*-value**
Gestational age (week)	0.928 (0.842–1.022)	0.129	-	-
IVF conceptions	3.163 (2.120–4.721)	<0.001	2.604 (1.719–3.944)	<0.001
BMI (kg/m^2^)	1.163 (1.113–1.215)	<0.001	1.151 (1.100–1.204)	<0.001
TBA (μmol/L)	1.061 (1.004–1.122)	0.035	-	-
A/G	1.313 (0.665–2.589)	0.433	-	-
TBIL(μmol/L)	1.001 (0.949–1.055)	0.977	-	-
DBIL (μmol/L)	1.068 (0.904–1.263)	0.440	-	-
IBIL (μmol/L)	0.992 (0.928–1.061)	0.820	-	-
PAB (mg/L)	1.010 (1.005–1.015)	<0.001	1.006 (1.001–1.011)	0.024
TP (g/L)	1.018 (0.976–1.062)	0.398	-	-
Albumin (g/L)	1.042 (0.985–1.102)	0.151	1.067 (1.005–1.133)	0.033
Globulin (g/L)	0.993 (0.945–1.044)	0.792	-	-
AST (U/L)	1.011 (1.005–1.017)	<0.001	-	-
ALT (U/L)	1.007 (1.004–1.010)	<0.001	1.007 (1.004–1.011)	<0.001
ALP (U/L)	1.001 (0.998–1.003)	0.510	-	-
γ-GT (U/L)	1.019 (1.010–1.028)	<0.001	-	-
LDH (U/L)	1.004 (1.000–1.008)	0.042	-	-
chE (U/L)	1.003 (1.000–1.006)	0.029	-	-
FBG (mmol/L)	1.750 (1.334–2.295)	<0.001	-	-

### Test Failure Rate Was Higher in IVF Conceptions

**Figure 2:**
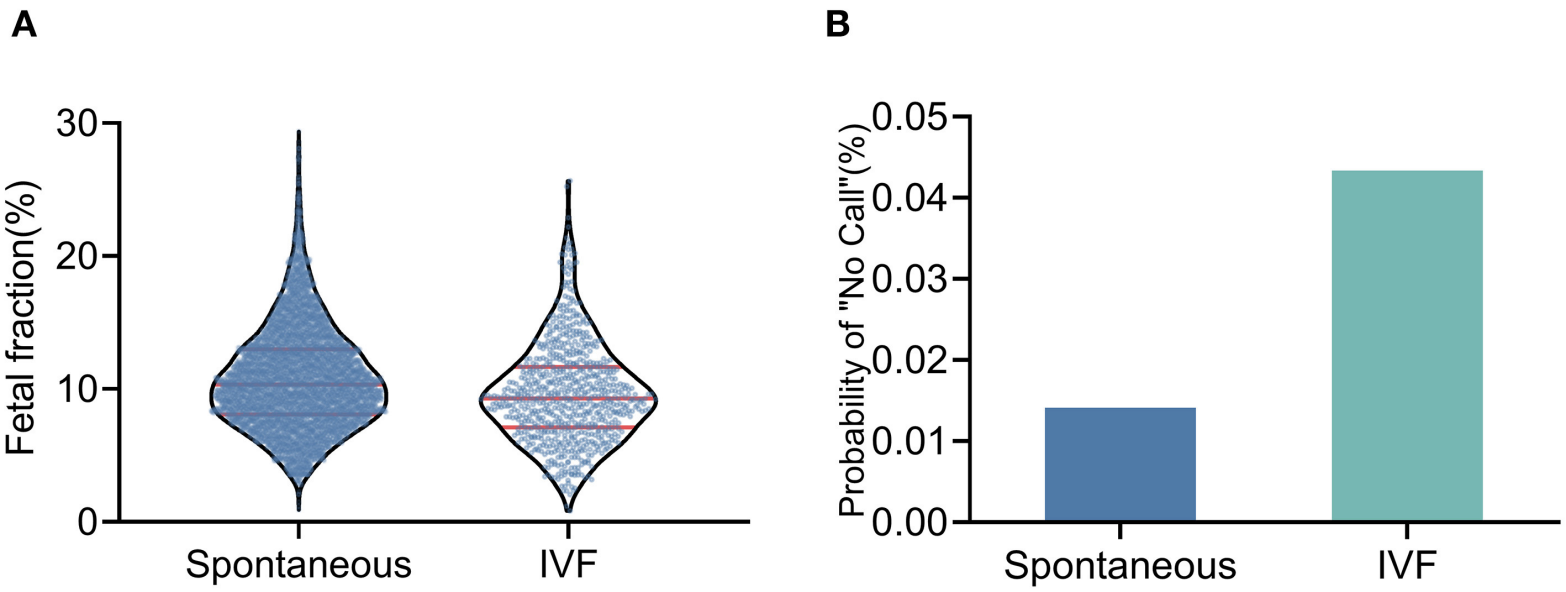
Fetal fraction and test failure rate of NIPT in IVF conceptions and spontaneous conceptions **(A)** The median fetal fraction was lower in IVF pregnancies. **(B)** The test failure rate was higher in IVF pregnancies.

The IVF conceptions had a lower median FF than spontaneous conceptions (9.29 vs. 10.33%, *P* < 0.001; Figure 2A); these differences remained statistically significant after adjusting for GA and BMI. The test failure rate was significantly higher in pregnancies conceived by IVF than in spontaneous conceptions (4.34 vs. 1.41%, *P* < 0.001; Figure 2B); these differences remained statistically significant after adjusting for BMI and ALT, PAB, and albumin levels (Table 2).

### Test Failure Rate Was Higher With Increasing ALT

**Figure 3:**
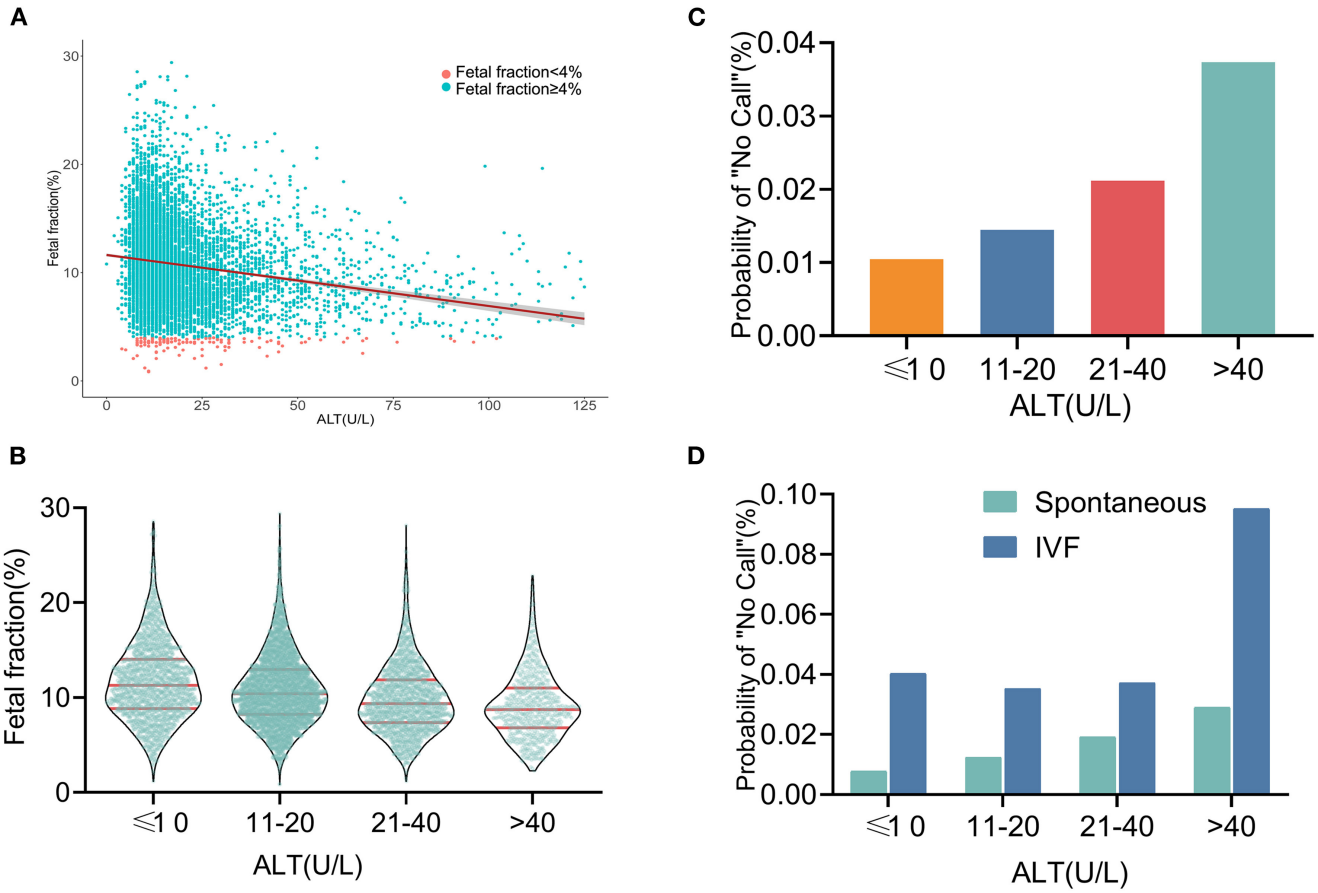
Relationship between fetal fraction and ALT **(A)** Scatterplots of fetal fraction according to ALT levels with lines of best fit from linear regression. **(B)** Mean fetal fractions across ALT categories. **(C)** Test failure rate according to different groups of ALT. **(D)** In the same ALT categories, the probability of “no call” rates was significantly higher in IVF conceptions than in spontaneous conceptions. Similarly, in the spontaneously conceived pregnancies, the probability of “no call” rates were higher in the higher ALT group, especially in the group with an ALT level of >40 U/L.

**Figure 4:**
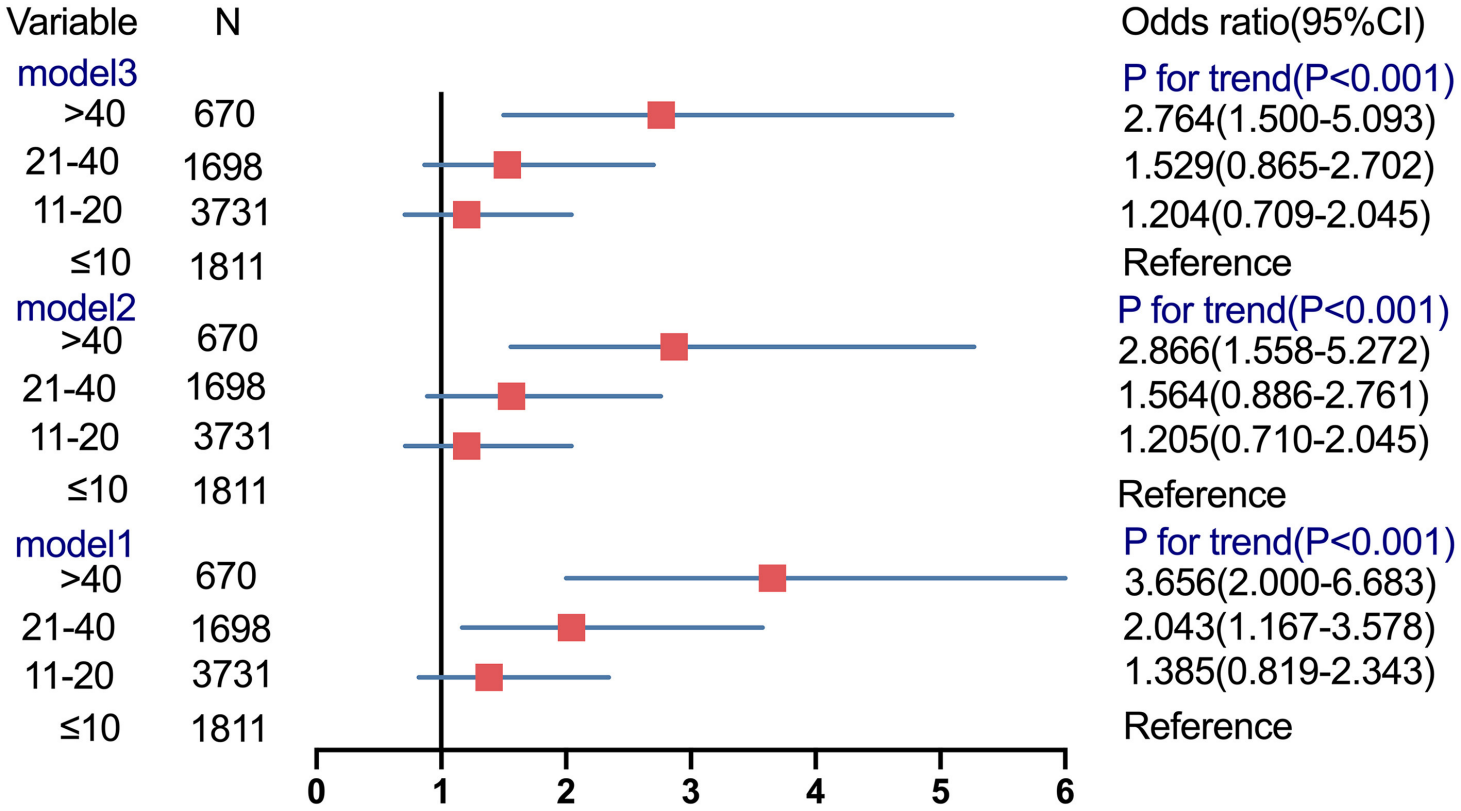
Association between different groups of ALT and risk of cfDNA screening failures.

As shown in Figure 3A, the ALT level was negatively correlated with the FF in the NIPS. To facilitate clinical application, the ALT levels were categorized as ≤10, 10–20, 21–40, and >40 U/L. The number of pregnant women with ALT in these four categories were 1,811, 3,731, 1,698, and 670, respectively. The mean FF across the ALT categories was 12.60% (95% CI, 12.34–12.87%), 11.55% (95% CI, 11.37–11.72%), 10.33% (95% CI, 10.09–10.58%), and 9.46% (95% CI, 9.10–9.83%) (Figure 3B), suggesting that the high ALT with low FF may increase the test failure rate. Although they were still low, the screening failure rates increased with increasing ALT levels from 1.05% at ≤10 U/L to 3.73% at >40 U/L (Figure 3C). The above-mentioned results suggest that IVF conceptions are an important factor affecting the test failure rate. Subsequently, a multivariable analysis was performed after controlling for IVF conceptions, and the results were shown using the same ALT categories. The probability of the “no call” rates was significantly higher with IVF conceptions than with spontaneous conceptions. Similarly, in spontaneously conceived pregnancies, the probability of the “no call” rates was higher in the higher ALT group, especially in the group with an ALT level of >40U/L (9.52%; Figures 3D, 4).

We also constructed three models to further test the independent association between the ALT categories and risk of cfDNA test failures (Figure 4). In the unadjusted Model 1, the odds of a test failure were >1 only among women with ALT levels of 21–40 U/L and >40 U/L (OR, 2.0434; 95% CI, 1.167–3.578 and OR, 3.656; 95% CI, 2.000–6.683, respectively). Moreover, compared with an ALT level of ≤10 U/L, the multivariable-adjusted OR (Model 3) of the test failures still increased significantly with increasing ALT levels of >40 U/L (OR, 2.764; 95% CI, 1.500–5.093; *P*_*trend*_ <0.001).

**Figure 5:**
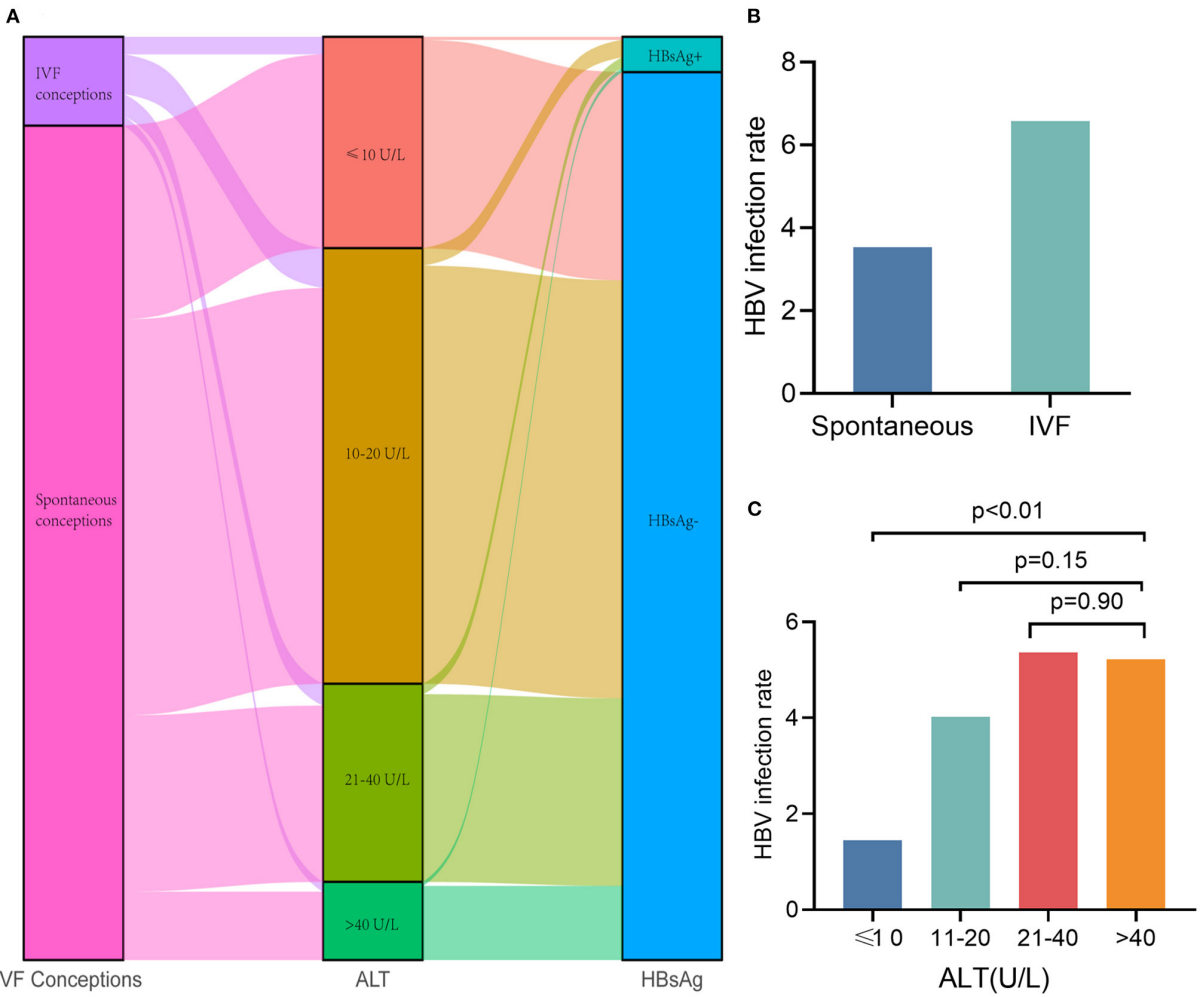
Hepatitis B infection does not affect NIPS test failure. **(A)** Sankey diagram showing sample conception pattern, ALT categories and HBV infection. **(B)** The HBV infection rate in IVF conceptions was significantly higher than that in spontaneous conceptions. **(C)** The HBV infection rates with ALT categories were 1.43, 4.02, 5.36, and 5.22%, respectively.

It is of great interest to clinicians whether the high infection rate of Hepatitis B virus (HBV) in the Chinese population and influence of liver function are caused by increase in ALT and NIPS test failure. We found that the prevalence of HBV infection among pregnant women in this study was 3.82%, which was slightly lower than the 5.05% reported among women in a previous systematic review (24). We also found that the HBV infection rate in IVF conceptions was significantly higher than that in spontaneous conceptions (Figures 5A,B). The HBV infection rates with ALT categories were 1.43, 4.02, 5.36, and 5.22%, respectively (Figure 5C). It has been suggested that HBV infection is not the reason for the test failures caused by elevated ALT levels, especially the test failures with a significant effect of >40 U/L. Therefore, we added HBV infection to the above Model 3. The result suggested that HBV infection has no effect on the NIPS test failure (OR, 0.773; 95% CI, 0.279–2.139; *P* = 0.62).

## Discussion

A low FF can lead to false negative results and test failures, thereby affecting the reliability of NIPS results. Therefore, it is important to investigate which candidate factors are associated with the FF and test failure rates. Our results indicated that increased BMI, ALT, PAB, and albumin levels, and IVF conception rate were independently associated with screening failures. The test failure rate was higher (4.34 vs. 1.41%; *P* < 0.001) in IVF pregnancies than in pregnancies by spontaneous conception. In particular, IVF pregnancies with an ALT level of >40U/L had a higher test failure rate (9.52%), indicating that pre-test counseling should be provided for women with such pregnancies.

Previous studies suggested that the IVF conception rate and high BMI were significantly associated with a lower FF, which may increase test failures (25). We also found that the risk of test failure increased with increasing BMI and in the cases of IVF conceptions. In particular, the factors affecting the FF reduction of IVF conceptions attracted our attention. Earlier, researchers speculated that the lower FF in IVF conceptions may be a consequence of smaller placental mass (25). However, compared with weight and thickness of the placentas from spontaneous pregnancies, those from IVF conceptions were significantly higher (26). More importantly, placental dimensions or weight had no impact on cfDNA levels in pregnancies by other observations, and this speculation has been refuted by other investigators (27). Recently, Herman et al. (28) explored the relationship between placental function and pathogenesis for these adverse outcomes associated with IVF treatments, proposing that villitis of unknown etiology, which results in shallow placentation, was significantly more common in the IVF group; therefore, reduced FF in IVF conceptions may be a consequence of shallow placentation.

Another possible explanation could be that cfDNA of maternal origin increases, causing a relative reduction in the FF. There is some robust evidence that NIPS test failures are higher in IVF conceptions than in spontaneous conceptions (25); IVF increases liver damage when the women are treated with high-dose gonadotropins (29). In addition, hormonal therapies, including oral contraceptives, in women with IVF-conceived pregnancies, may impair glucose and lipid metabolism, promote insulin resistance and inflammation, and increase maternal cfDNA release, resulting in decreased FF (30). Interestingly, our previous research found that the FF and the rate of NIPS are affected by triglyceride (TG) levels (12). In this study, we also found that the TG levels in case of IVF conceptions were significantly higher than those in spontaneous pregnancies (Supplementary Figure). Moreover, the TG levels showed a strong positive association with leukocyte count. CfDNA released by leukocytes accounts for 70% of the total cfDNA (15, 31). Therefore, we speculated that higher TG levels in IVF conceptions might affect the test failure rate by means of their role in leukogenesis. With progressive inflammation and endothelial damage, cfDNA of maternal origin may be released more readily into the circulation. Recent studies have demonstrated that the test failure rate was significantly increased in patients with preeclampsia, which is in accordance with the possibility of shallow placentation and inflammation in these cases (32).

To the best of our knowledge, our results demonstrated for the first time that higher levels of ALT, PAB, and albumin may be closely related to increasing rates of screening failures. This association could be attributed to the increase in the cfDNA released from the maternal liver. Hepatocyte-derived cfDNA, released from dying hepatocytes, correlated with the level of ALT, which is reflective of liver damage causing its release into the blood (33). Additionally, maternal-derived DNA from liver is the major source of cfDNA in maternal plasma. In particular, more liver-derived cfDNA will be released when the liver is damaged during pregnancy. We found a lower median FF and a higher screening failure rate in the liver damage group (ALT level > 40 U/L), and these differences remained statistically significant after adjusting for confounders. We also explored whether factors affecting ALT elevation, such as HBV infection, affect the NIPS test failure rate. In the group with ALT levels >20 and 40 U/L, there was no difference in the HBV infection rate, and the NIPS test failure rate increased with increase in the ALT level, which may indicate that other diseases of pregnancy affect ALT and promote the liver to release more maternal-derived cfDNA. Moreover, we added HBV infection to the above Model 3 and found that HBV infection did not affect the NIPS test failure rate.

This study has some limitations, mentioned as follows. The study did not explore the factors affecting liver function and the duration required to significantly reduce NIPS test failure after normal liver function is achieved. These points will be the focus of our future research. Nevertheless, to the best of our knowledge, this is the first study to identify the relationship between liver enzymes and the test failure rate, adjusted for a number of potential confounders in cfDNA tests. Increased ALT level and IVF conception rate were associated with higher NIPS failure rates, suggesting that women with pregnancies through IVF conception who choose NIPS for aneuploidy need to be aware of a higher testing failure rate, especially if they have an underlying liver damage. Delaying the cfDNA test while reducing liver damage may benefit these pregnant women. However, this strategy may increase the waiting time for making timely decisions and may increase maternal anxiety. Furthermore, alternative screening by ultrasonography, size-selection NIPS (sequencing shorter cfDNA, which significantly improves the FF), or offering diagnostic testing is recommended after screening failures.

## Data Availability Statement

The datasets for this article are not publicly available due to concerns regarding the anonymity of the participant/patient. Requests to access the datasets should be directed to the corresponding author, and appropriate reasons should be provided. Requests to access the datasets should be directed to YL, e-mail: liangyuting666@126.com.

## Ethics Statement

Written informed consent was obtained from the individual(s) for the publication of any potentially identifiable images or data included in this article.

## Author Contributions

PC, LQ, SZ, and JJ: conception, design, collection, and assembly of data. JC and YZ: administrative support. HT, ZY, and JS: provision of study materials or patients. JY, YL, and XW: data analysis and interpretation. All authors: writing and final approval of the manuscript.

## Funding

This study was supported by the National Natural Science Foundation of China (Grant Nos. 82001576 and 81901632); Suzhou Science and Technology Support Program (SYS2019095, SYS2019098, BE2019683, and SS2019066); Jiangsu Science and Technology Support Program (SBE2019740167); Suzhou Clinical Medical Expert Team (SZYJTD201708); and Jiangsu Provincial Medical Innovation Team (CXTDB2017013).

## Conflict of Interest

The authors declare that the research was conducted in the absence of any commercial or financial relationships that could be construed as a potential conflict of interest.

## Publisher's Note

All claims expressed in this article are solely those of the authors and do not necessarily represent those of their affiliated organizations, or those of the publisher, the editors and the reviewers. Any product that may be evaluated in this article, or claim that may be made by its manufacturer, is not guaranteed or endorsed by the publisher.
