# Supplementary material for: The Effect of Elevated Alanine Transaminase on Non-invasive Prenatal Screening Failures
Source: Front Med (Lausanne). 2022 Jun 15;9:875588. doi: 10.3389/fmed.2022.875588 (PMC9240308; doi:10.3389/fmed.2022.875588)

Supplemental figure. The triglyceride levels were higher in IVF pregnancies than in spontaneous conceptions.


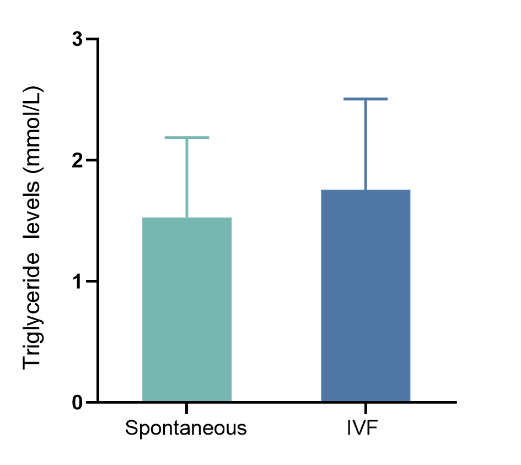

Supplement: Supplementary file 1 [file Table_1.DOCX]
